# Comprehensive experimental and theoretical investigations of novel triazine Schiff base metal complexes: spectroscopic, electrochemical, DNA interaction, in vitro cytotoxicity, antimicrobial, and in silico studies

**DOI:** 10.1186/s13065-026-01761-w

**Published:** 2026-03-18

**Authors:** Gehad Abd El-Hakeem, Gamal A. Gouda, Khaled A. Abd El-Rahem, Shimaa Hosny

**Affiliations:** 1 Department of Chemistry, Faculty of Science, Assiut Branch, Al-Azhar University, Assiut, 71524 Egypt; 2 Department of Chemistry, Faculty of Science, New Valley University, Alkharga, 72511 Egypt

**Keywords:** Schiff base, DNA interaction, Cytotoxicity, antimicrobial activity, cyclic voltammetry, Computational

## Abstract

The increasing resistance of bacteria and cancer to present treatments is a clinical and laboratory concern. In this study, a novel Schiff base ligand (HL) was synthesized in this work through a solvent-free method, involving the reaction of 2,4-diamino-6-phenyl-1,3,5-triazine with 2-hydroxy-1-naphthaldehyde under mild and eco-friendly conditions. The Ni(II) and Zn(II) complexes were then synthesized and confirmed by different spectroscopic and analytical methods. The results suggest that both complexes have an octahedral geometry, in good agreement with the computational findings. The binding mood of these compounds with CT-DNA was examined using cyclic voltammetry and UV-vis spectroscopy, which revealed a partial intercalation mode. The findings demonstrated strong DNA-binding affinity with binding constants (K_b_) of 2.51 × 10^5^ and 5.69 Χ 10^5^ M^− 1^ for NiL_2_ and ZnL_2_ complexes, respectively. The calculated free energy (ΔG) values confirmed that these interactions with DNA are strong and spontaneous. Additionally, the compounds’ anticancer efficacy was tested against HepG-2 liver cancer cells. IC_50_ values for HL, NiL_2_, and ZnL_2_ were calculated and determined to be (94.10, 51.08, 43.94) µg/mL. This shows that these complexes can preferentially treat liver cancer. The tested microorganisms demonstrated better response to metal complexes compared to the Schiff base ligand. Finally, molecular docking studies were performed to determine the binding interactions and affinities of the prepared compounds with the active site of the receptors associated with liver cancer (5A19) and *A. flavus* (8HBS). The study revealed adding metal ions considerably raised molecular affinities, and the binding potency sequence was ZnL_2_> NiL_2_ > HL ligand. According to experimental and theoretical evidence, the novel prepared ligand and its metal complexes have great promise as multifunctional bioactive agents, enabling additional pharmacological and biological applications.

## Introduction

Schiff bases are chemical compounds with azomethine (–C = N–) groups. A primary amine and a reactive carbonyl molecule like an aldehyde or ketone condense to create them [1–4]. Schiff bases are of significant importance due to the presence of the carbon-nitrogen double bond (C = N), which allows them to coordinate with metal ions [5–8]. These versatile compounds exhibit diverse biological activities, including analgesic, anti-inflammatory, antibacterial, antifungal, antioxidant, antitumor, cardiovascular, and antitubercular properties, as well as local anesthetic effects [9, 10]. Schiff bases derived from hydroxyl aldehydes, which include both oxygen and nitrogen donor sites, can coordinate with central metal ions to form metal complexes [11, 12]. These metal complexes have received a lot of interest due to their numerous uses in various fields [13–15]. Notably, such complexes exhibit strong DNA intercalation and promising biological activity, making them highly significant in medicinal chemistry. Among biologically active Schiff bases, 2,4-diamino-6-phenyl-1,3,5-triazine is particularly important due to its potent bioactivity. Numerous structurally novel triazine derivatives have been reported as promising lead compounds for the development of new antitumor agents [16]. Triazine compounds and their derivatives have a wide range of pharmacological activities, including antibacterial, antiviral, antimalarial, and anti-inflammatory effects, as well as potential anticancer, antileukemic, and anti-HIV properties [17, 18]. Furthermore, triazine analogs are crucial components for creating multisite ligand complexes [19, 20]. Over the past few years, the potential applications of triazine derivatives in agrochemistry and medicine have been extensively investigated [21]. The ongoing search for effective anticancer treatments has put a lot of focus on the development of novel metal-based medicines [22]. Although chemotherapeutic drugs based on platinum have shown promise in treating many solid tumors by causing cancer cells to die off [23], their clinical success has been limited by issues such as drug resistance, reduced efficacy, and severe side effects on healthy tissues [24]. Consequently, there is growing interest in the design of transition metal-based drugs that selectively target cancerous DNA while sparing normal cells [25]. In this context, the selection of transition metals coordinated with biocompatible ligands is crucial [26]. Schiff base ligands, which consist of several nitrogen and oxygen atoms, have recently gained interest for their ability to chelate metals in a variety of ways and their wide range of biological activities, which include antibacterial, antiviral, and anticancer effects [27]. To utilize Schiff base metal complexes as structural probes for nucleic acids, one must have a thorough understanding of the chemical interactions between DNA and these complexes. You may change the way metal complexes interact with DNA by changing their redox characteristics, ligand structures, and the physiological functions of metal ions [28]. Nickel (II) and zinc (II) will be used in this investigation because of their well-established anticancer properties and minimal damage to healthy cells. Reactive oxygen species (ROS), such as superoxide anions and other radicals, are natural byproducts of aerobic respiration and can harm essential biomolecules [29].

The creation and analysis of new Schiff base ligand and their metal complexes are detailed in this work. Furthermore, we investigate their pharmacological properties, including cytotoxic and antimicrobial activities, as well as their CT-DNA binding affinity. Molecular docking studies were conducted against selected protein targets (PDB ID: 5A19 and PDB ID: 8HBS) to further validate their biological activities. The structural stability of the ligand and its metal complexes was further guaranteed through optimization utilizing DFT/B3LYP/LANL2DZ basis set calculations.

## Experimental

### Chemicals used and physical measurements

All chemicals used in this study were of analytical reagent grade and were employed without further purification. The following materials were obtained from Sigma-Aldrich: 2,4-diamino-6-phenyl-1,3,5-triazine, 2-hydroxy-1-naphthaldehyde, ethanol, NiCl₂·6 H₂O, and ZnCl₂·2 H₂O.

Elemental analyses (CHNS) were performed using a Perkin-Elmer 240 C elemental analyzer. Melting points of the synthesized compounds were determined using a Gallenkamp melting point apparatus. Fourier-transform infrared (FT-IR) spectra were recorded in the range of 4000–400 cm⁻¹ using a Shimadzu spectrophotometer with KBr pellets. Molar conductance measurements were carried out using a Jenway conductometer. Electronic absorption spectra were measured using a Jasco P-530 UV-Vis spectrophotometer. Proton nuclear magnetic resonance (^1^H NMR) spectra were recorded in DMSO-d₆ on a Bruker 400 MHz NMR spectrometer. Thermogravimetric analysis (TGA) of both micro- and nanocomplexes was conducted under an inert atmosphere using a Shimadzu DTG-60 H thermal analyzer at a heating rate of 10 °C/min. Powder X-ray diffraction (PXRD) patterns were collected using a Bruker AXS D8 Advance diffractometer. Scanning electron microscopy (SEM) images were obtained using a Jeol JEM-1200 EX II microscope operated at an accelerating voltage of 25 kV. Cytotoxicity assessments were performed using a Metertech Σ960 ELISA microplate reader.

### Eco-friendly synthesis of Schiff base (HL)

**Fig. 1:**
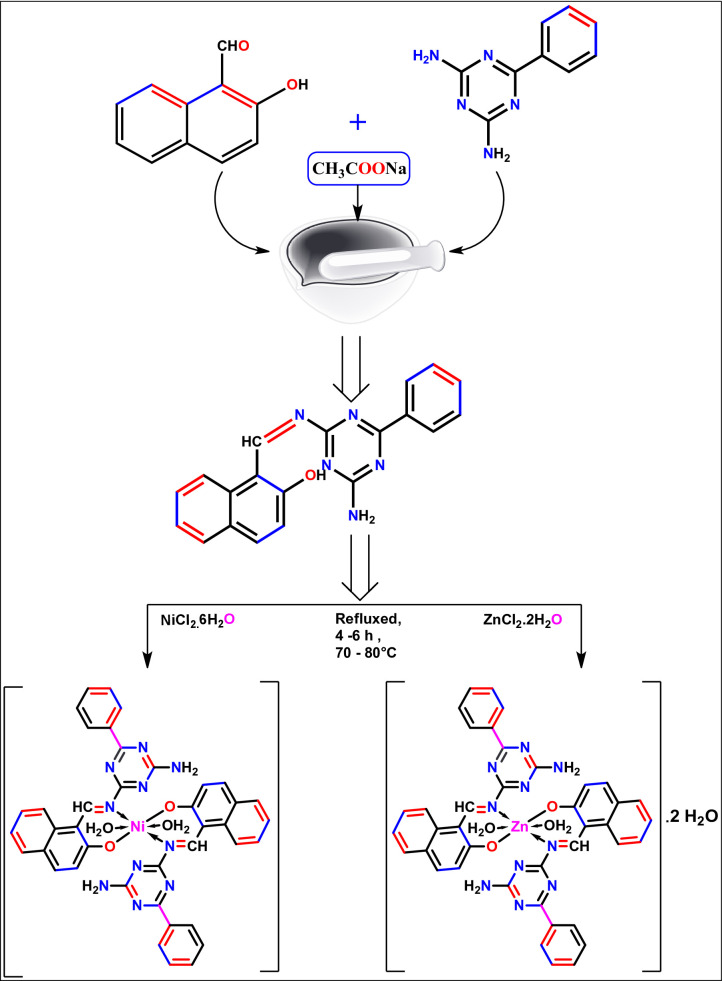
Illustrates an eco-conscious synthetic method for creating the Schiff base ligand (HL) and its corresponding metal complexes

Accurately quantify equimolar proportions of 2,4-diamino-6-phenyl-1,3,5-triazine and 2-hydroxy-1-naphthaldehyde in the presence of anhydrous sodium acetate. Meticulously amalgamate the reagents utilizing a mortar, subsequently grinding the reaction mixture while sustaining a temperature range of 40–80 °C to promote the condensation reaction. Persist in grinding until a solid product emerges, signifying the successful synthesis of the Schiff base. The resultant solid is then solubilized in distilled water to eliminate sodium acetate through the process of filtration. The acquired precipitate is collected, refined through recrystallization from ethanol, and ultimately dehydrated under vacuum conditions (Fig. 1).

### Synthesis of complexes

To produce metal chelates, the HL ligand mixture was refluxed in 20 milliliters of hot ethanol with a 1:1 molar ratio solution of ZnCl_2_.2H_2_O or NiCl_2_.6H_2_O. After four to six hours of reflux at 70 °C, the reaction mixtures were filtered, washed with ethanol, and dried at room temperature (Fig. 1).

### Theoretical calculation

Gaussian 09 was used to optimize the produced compounds’ molecular structures using DFT simulations [30–32]. The LANL2DZ basis set was employed for metal centers (Ni and Zn) and the B3LYP functional and 6-311G basis set for non-metal atoms.

### DNA binding

#### UV–visible spectroscopic method

UV-visible spectroscopy was used to study the interaction between metal complexes and CT DNA in a pH 7.0 Tris-HCl buffer. The concentration of CT DNA was calculated using a molar extinction value of 6600 M^− 1^ cm^− 1^ and absorbance at 260 nm. The compounds were dissolved in 4% DMSO for better solubility before dilution.

#### Cyclic voltammetry (CV) method

A VersaSTAT 4 potentiostat/galvanostat manufactured by Princeton Applied Research in the USA was used to conduct cyclic voltammetry (CV) measurements. Three electrodes made up the electrochemical cell: a reference electrode (K0265) made of Ag/AgCl, a counter electrode (K0266) made of platinum wire, and the working electrode (GPE) made of graphite pencil. A scanning electron microscope (SEM, JEOL JSM-5400 LV) was used to examine the graphite powder’s surface morphology, while FT-IR spectroscopy and X-ray diffraction (XRD) provided structural details. The graphite powder used to create the GPE was characterized using FT-IR, XRD, and SEM methods before electrochemical studies were conducted. A rotring tikky special pencil (Germany, 0.5 mm) fitted with a 2B graphite lead (0.5 mm diameter) was used as the working electrode. The pencil was positioned vertically. A length of 4 mm of the graphite lead was left outside the holder, while 6 mm was immersed in the electrolyte solution. CV measurements were performed with a K0264 Micro-Cell Kit. The cell contained 10 mL of the test solution. Nitrogen purging for 15 min was applied before measurements to eliminate dissolved oxygen. Accumulation was carried out on the pencil graphite electrode at an accumulation potential of 0 V for 30 s under stirring. The scan rate was set to 100 mV s⁻¹. After the accumulation step, stirring was stopped and a quiet time of 15 s was allowed before starting the potential scan.

#### Chemicals and solutions

ct-DNA (calf thymus) was obtained from Sigma. No purification was performed. DNA stock solution was prepared in distilled water and stored at 4 °C. DNA concentration was 5.45 × 10⁻⁵ M (nucleotide phosphate basis). The value was determined from UV absorbance at 260 nm using ε = 6600 M⁻¹ cm⁻¹. Under the experimental conditions, the analyte concentration did not change within 12 h. Phosphate buffer was used as the electrolyte. All reagents were analytical grade (Merck). Double-distilled water was used. pH was measured using a Jenway 3310 pH meter (± 0.02).

#### Characterization of graphite pencil electrode

**Fig. 2:**
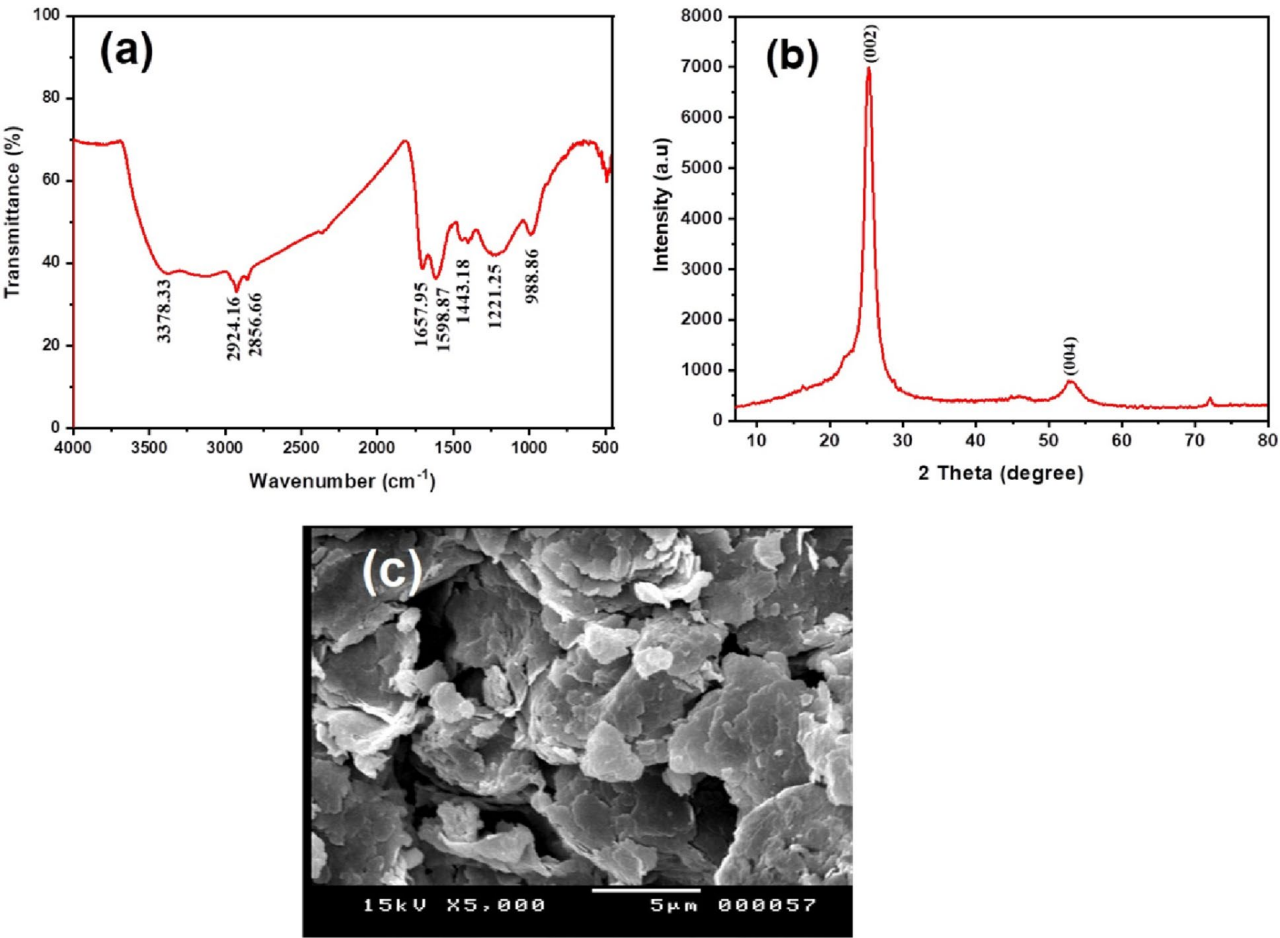
Spectra characterization of graphite nanoparticles: FT-IR spectrum **a**, ⅩRD **b**, SEM image **c**

Figure 2(a) shows the FT-IR spectrum of graphite nanoparticles. Th Special peaks at 2924.16 and 2856.66 cm^− 1^ owing to the graphite carbon stretching vibration mode, the broad absorption band centered at 3378.33 cm^− 1^ is assigned to O–H stretching vibrations, and the band at 1657.95 cm^− 1^ is attributable to H–O–H bending vibration mode. The graphite was characterized by XRD in Fig. 2(b). Well-defined diffraction peaks are observed in the two peaks at 2θ = 26.25° and a slight peak at 2θ = 54.4° was observed specific to the (002) and (004) planes, which are in accordance with the standard spectrum (JCPDS, No. 41-1487, hexagonal). The average particle size of the sample was found to be 17 nm. which is derived from the full width at half-maximum (FWHM) of more intense peaks corresponding to the (002) plane located at 26.25° using Scherrer’s Eqs [33, 34]. The surface morphology of the graphite was examined by SEM images; Fig. 2(c) shows the SEM image of graphite nanoparticles with a magnification of 5000.

#### Antimicrobial activity

The disc diffusion method [35–37], was applied to determine the antibacterial and antifungal activity of the produced compounds. The study included fungal strains as well as bacterial strains. Test chemicals were dissolved in DMSO and added to 5 mm sterile discs on nutrient agar plates at a concentration of 100 ppm. After 24 h of incubation at 37 °C, the inhibition zones (IZ) were evaluated to determine activity. The results were compared to common antimicrobial drugs, including clotrimazole (CLO) for fungi and chloramphenicol (CHL) for bacteria.

#### Cytotoxicity study

Tumor cell lines were grown at 37 °C and 5% CO₂ in RPMI-1640 media with 10% fetal calf serum and gentamycin. In 96-well plates, cells (5 × 10⁴ per well) were plated and treated in triplicate with ten different drug dosages. In control wells, just DMSO or media were present. The MTT test was performed to determine cell viability after a 24-hour treatment period. The percentage of living cells was calculated in comparison to untreated controls using absorbance measurements at 590 nm. GraphPad Prism software was used to obtain IC₅₀ values from dose-response curves [38–40].

#### Molecular docking studies

Docking investigations were conducted utilizing MOA2022 software [41], to determine how the synthesized substances interact with key biological targets. The docking simulations were designed to identify the binding affinities and interaction patterns inside the active regions of two receptors: the liver cancer-associated protein (PDB ID: 5A19) [42], and a gene regulation protein from *A. flavus* (PDB ID: 8HBS) [43].

## Results and discussion

### Conductometric analysis and elemental assessment

Metal (II) complexes exhibit molar conductance values ranging from 6.3 to 11.5 ohm^− 1^ cm² mol^− 1^, indicating their non-ionic nature, and hence they are considered as non-electrolytes [44]. The proposed chemical formulas C_20_H_15_N_5_O for the HL, C_40_H_32_N_10_NiO_4_ for the NiL_2_, and C_40_H_36_N_10_O_6_Zn for the ZnL_2_ are highly supported by the complexes’ elemental analysis data, which closely match the computed theoretical values (Table 1). A remarkable degree of agreement between the predicted and experimental elemental compositions validates the excellent stoichiometry and purity of the synthesized complexes. Overall, our research demonstrates that the complexes are neutral, stable coordination molecules that do not exhibit electrolytic activity in DMSO solution. The strong match between the observed and anticipated elemental data supports the proposed structural formulas and shows that the metal centers and HL ligand are well-coordinated.

**Table 1:** Compilation of the physicochemical and analytical information to the compounds under investigation

Compound (Abbreviation)	Mol. Formula (M.wt)	%Yield	Color	M.*P*. (^o^C )	Ω_m_ (ohm^− 1^ cm^2^ mol^− 1^)	Elemental analysis %: found (calc.)			
						C	H	*N*	M
HL	C_20_H_15_N_5_O (432.50)	78	Yellow	175	–	70.42 (70.37)	4.41 (4.43)	20.58 (20.52)	–
[NiL_2_(H_2_O)_2_] (NiL_2_)	C_40_H_32_N_10_NiO_4_ (774.20)	67	Dark green	Dec. at 46	6.3	61.63 (61.96)	4.21 (4.16)	18.14 (18.06)	7.42 (7.57)
[ZnL_2_(H_2_O)_2_].2H_2_O (ZnL_2_)	C_40_H_36_N_10_O_6_Zn (674.11)	63	Dark brown	Dec. at 45	11.5	58.65 (58.72)	4.37 (4.44)	17.19 (17.12)	7.87 (7.99)

### Vibrational spectra

**Fig. 3:**
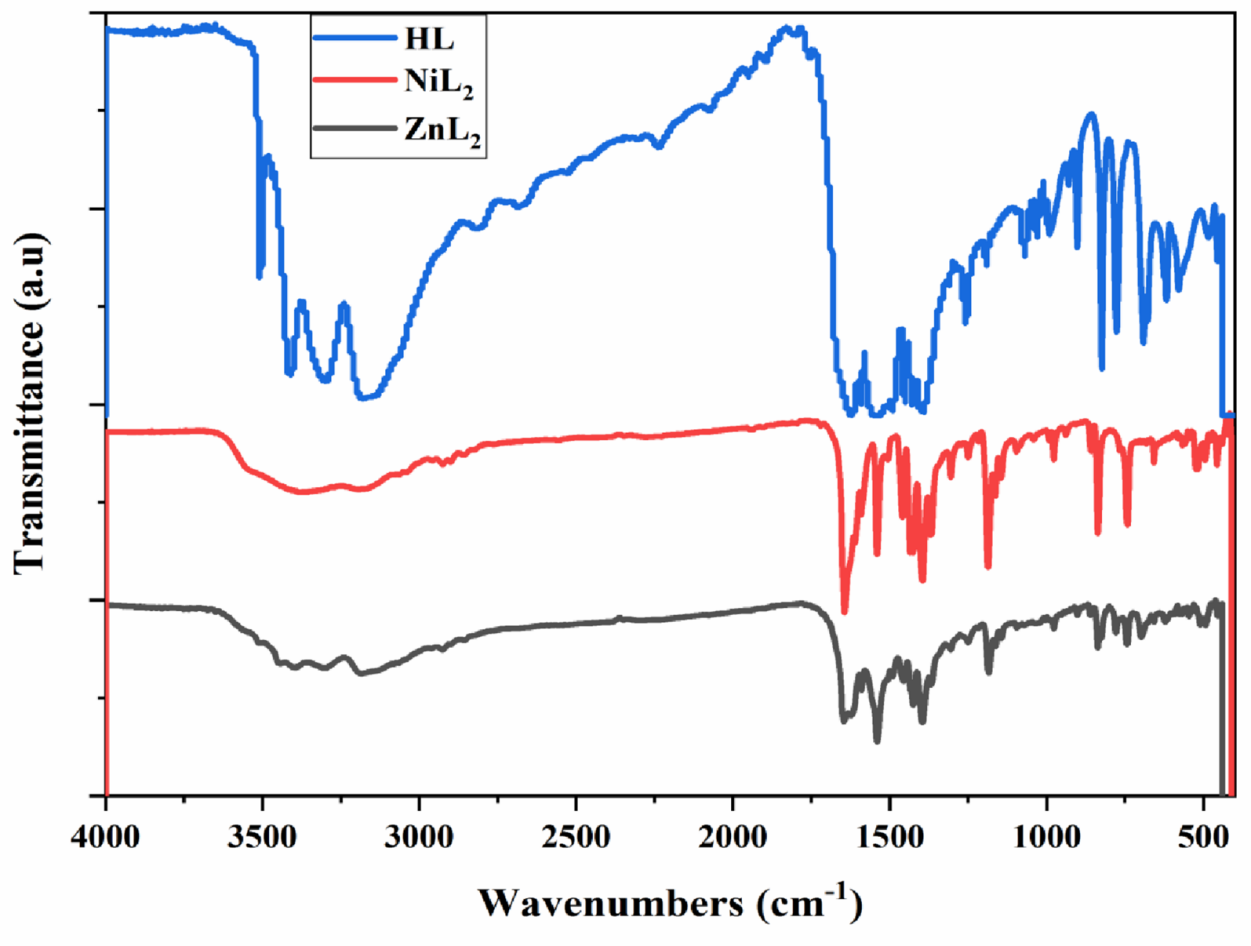
IR spectra of HL ligand and its NiL_2_ and ZnL_2_ complexes

The Schiff base and its metal complexes are provided with critical infrared spectrum information in (Fig. 3; Table 2). The Schiff base’s infrared spectra at 1630 cm^− 1^ exhibited a discernible absorption band, which represented the stretching vibration of the C = N bond. All of the complexes displayed the band of ν(C = N) in the range of 1612–1646 cm^− 1^ when compared to the Schiff base’s spectra, suggesting that the imine nitrogen atom is coordinated to the metal ion [45]. The ν(OH) group is responsible for the band that the Schiff base displays at 3310 cm^− 1^. In the NiL_2_ and ZnL_2_ complexes, this band disappeared, suggesting proton replacement during metal chelation. In metal complexes, the ν(NH₂) band, observed at 3170 cm^− 1^ [46], remained substantially unchanged, indicating that the amino group is not engaged in coordination. A broad band in the 3385–3397 cm^− 1^ area indicates the presence of coordinated water molecules. The triazine ring’s characteristic strong band, identified at roughly 1430 cm^− 1^ [47], was noticed in the spectra of metal complexes at the same positions, showing that (C = N) does not participate in coordination. A new bands is seen in the 513–517 and 453–457 cm^− 1^ region in the infrared spectra of the created complexes due to ν(M-N) and ν(M-O), respectively.

**Table 2:** IR data for the ligand (HL) and corresponding complexes

Compound	ν (C = *N*)	ν (O-H) phenolic	ν (NH_2_)	ν (C = *N*) triazine ring	ν(M-*N*)	ν(M-O)
HL	1630	3310	3170	1430	–	–
NiL_2_	1646	–	3170	1430	457	517
ZnL_2_	1612	–	3170	1430	453	513

### ^1^H-NMR spectra

**Fig. 4:**
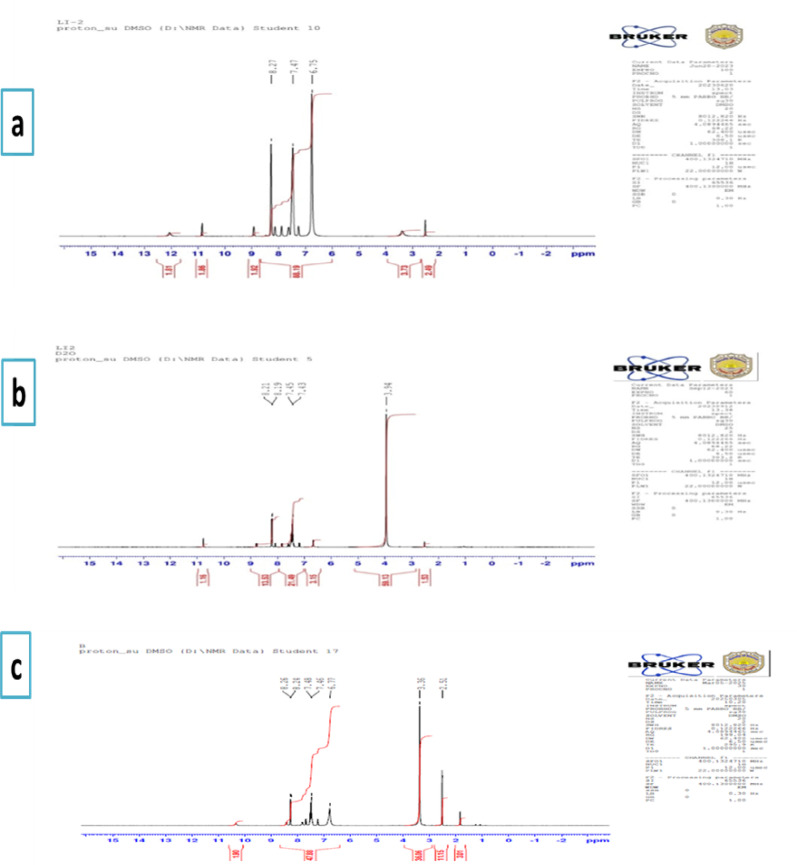
^1^ H NMR spectrum of **a** HL in DMSO **b** HL in D_2_O, and **c** ZnL_2_ in DMSO

The ^1^H-NMR spectrum of the ligand and its ZnL_2_ complex were achieved in DMSO-d6 and D_2_O (Fig. 4). The spectrum corresponding to the ligand exhibits distinct signals at δ 3.3, 6.75–8.27 ppm, 8.70, and δ 12.74 ppm, which are ascribed to the (s, NH₂), (m, aromatic protons), (s, azomethine proton), and (s, phenolic –OH proton) [48], respectively. The elimination of signals associated with the –OH and –NH_2_ protons upon D_2_O exchange substantiates the proposed assignments of signals within the ligand spectrum. In the spectra of Zn(II) complex, the retention of –NH₂ proton signals corroborates the non-involvement of this functional group in the coordination process. In Zn(II) complex, the azomethine proton signal is observed to shift up-field to δ 8.30 ppm, indicating a comparable interaction. Furthermore, the absence of the –OH (phenolic) signals in the spectra confirms the interaction of the metal ion with oxygen [48]. Since proton NMR data is unreliable due to the paramagnetic nature of the Ni(II) complex, the ¹H NMR spectrum was not obtained.

### Electronic and magnetic properties

**Fig. 5:**
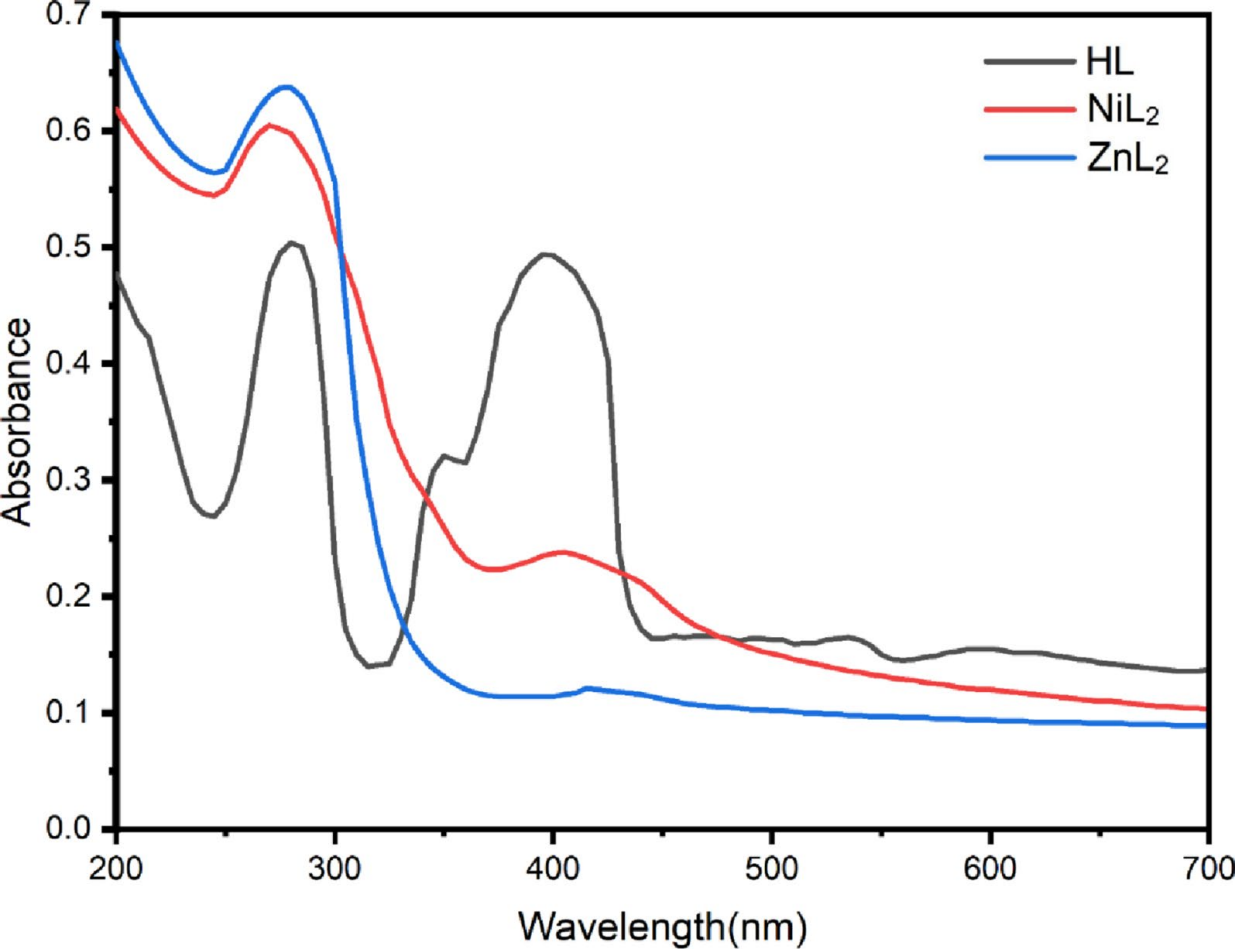
Electronic absorption spectra of the ligand (HL) and its corresponding metal complexes

Electronic absorption spectra of manufactured compounds disclose important electrical and structural features. Figure 5 shows the ligand (HL) and metal chelate spectra in DMSO. The HL exhibited two distinct bands. The initial detection, at λ_max_ = 280 nm, is due to π→π* transitions of aromatic rings. The second band, visible at 400 nm, corresponds to a change in charge transfer [49]. Three distinct absorption regions are present in the NiL₂ complex. A prominent band at 275 nm is the result of the π→π* transitions of the aromatic and azomethine moieties. The second band, which is observed at approximately 330 nm, is most likely the result of a metal-to-ligand charge transfer (MLCT). A third absorption band at approximately 405 nm is generated by a spin-allowed d-d transition. Additional d-d transitions are anticipated in the 758–802 nm and 988–1038 nm ranges, which correspond to the ³A₂g→³T₁g(F) and ³A₂g→³T₂g transitions, respectively. This is predicated on the ligand field theory and previously published results for octahedral Ni(II) complexes. The presence of these bands is indicated by a small tailing beyond 600 nm, despite the fact that they were not completely resolved in the current spectra [50]. The magnetic moment of the NiL_2_ complex was determined to be 2.93 B.M., confirming its octahedral geometry. The electronic spectra revealed three unique absorption bands in the ZnL₂ compound. The strong band at 275 nm is driven by π→π* transitions inside the ligand, which are commonly found in azomethine groups and aromatic rings. LMCT is connected with the second band, which is around 420 nm. Zn(II) does not have d-d transitions due to its fully filled d¹⁰ structure. Furthermore, the magnetic moment was measured and found to be 0 B.M., confirming an octahedral structure with no unpaired electrons and validating the diamagnetic behavior [51].

### Thermal analysis

**Fig. 6:**
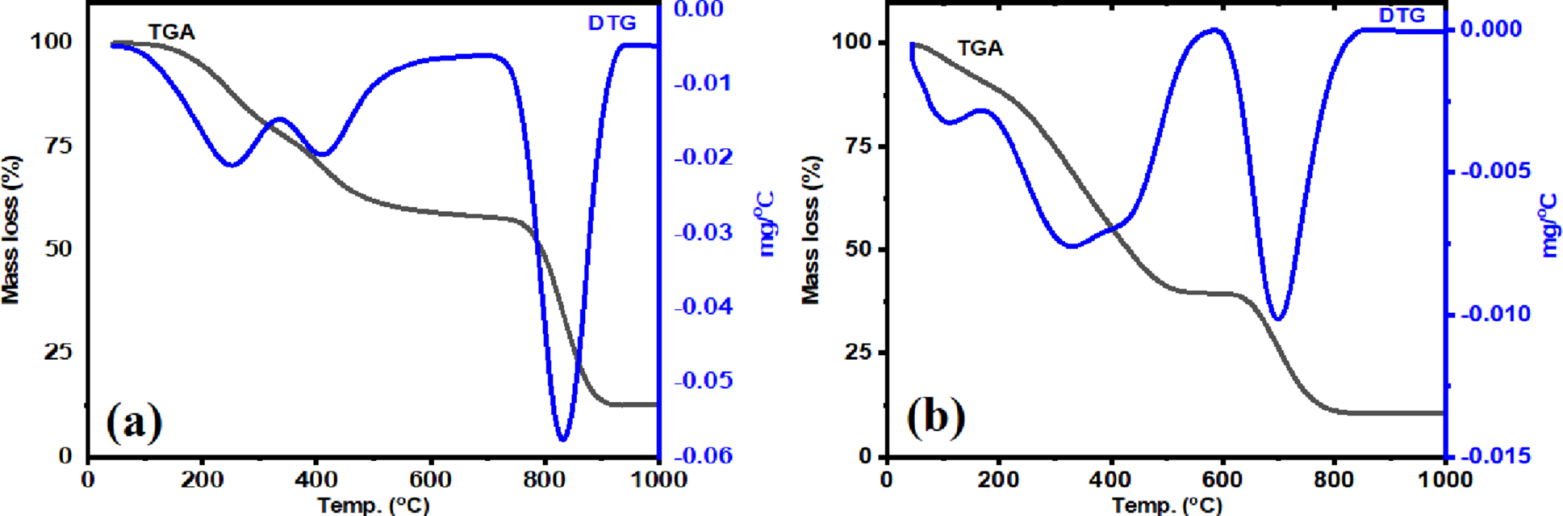
TGA/DTG curves of compounds NiL_2_ **a** and ZnL_2_ **b**

Thermogravimetric analysis (TGA) of the metal complexes was carried out from room temperature up to 1000 °C. The recorded mass losses occurred in successive steps consistent with the proposed compositions of the complexes. The first weight loss was attributed to the release of coordinated or hydrated water molecules, followed by decomposition of the organic parts of the complexes.As shown in Fig. 6(a), the Ni(II) complex decomposes in three steps. The first step takes place between 46 and 293 °C and corresponds to the loss of two coordinated water molecules together with two benzene units (2 H₂O + 2 C₆H₆) [2, 52]. The calculated mass loss for this step is 24.79%, while the experimental value is 24.15%. The second step occurs in the temperature range 338–654 °C and is assigned to the removal of two C₃H₃N₃ fragments, with a calculated loss of 20.94% and an observed loss of 20.42%. The third step extends from 655 to 989 °C and involves the decomposition of two C₁₁H₇NO units. The theoretical mass loss for this stage is 43.67%, compared with an experimental value of 43.95%. The final residue obtained after complete decomposition is NiO, representing 11.09% theoretically and 11.48% experimentally. In a similar manner, the Zn(II) complex (Fig. 6(b)) shows three decomposition steps. The first step, observed between 45 and 180 °C, is due to the loss of two coordinated and two hydrated water molecules (2 H₂O_coord_. + 2 H₂O_hyd.),_ giving an experimental mass loss of 8.95% (calculated 8.82%). The second step occurs between 180 and 581 °C and corresponds to the decomposition of 2 C₆H₆, 2 C₃H₃N₃, and 2HCN fragments, with a total mass loss of 51.60% (calculated 51.21%). The final stage, occurring between 581 and 896 °C, encompasses the degradation of the remaining 2 C₁₀H₆ fragment, with an experimental mass loss of 30.9% (calculated: 30.6%). The residual product comprises Zn and 0.5 C, with a theoretical mass percentage of 8.56% and an empirical mass percentage of 8.85%.

### DFT calculations

#### 3D optimization

**Fig. 7:**
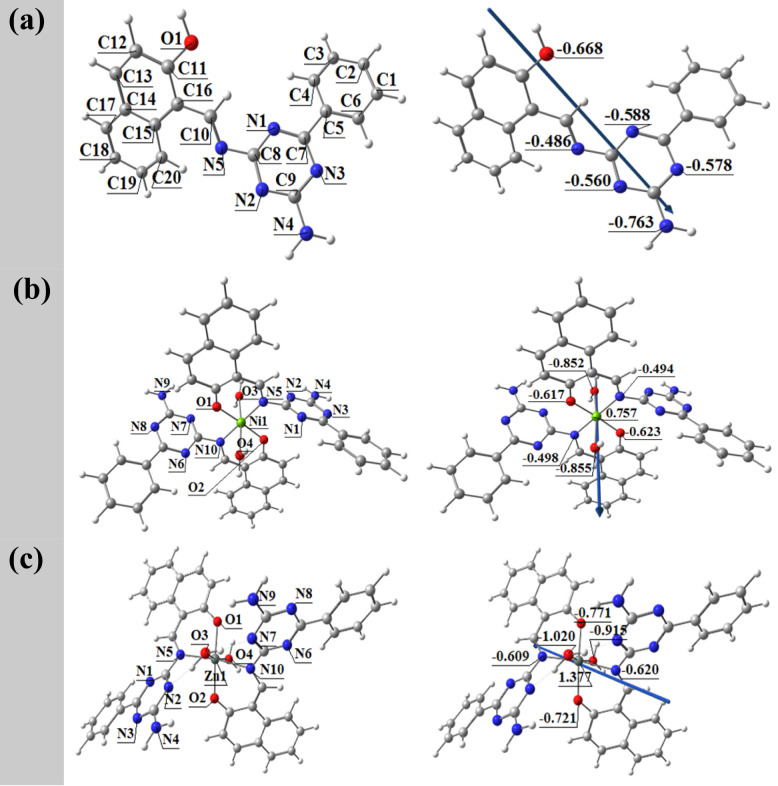
Optimized geometries, dipole moment vector, and natural atomic charges on coordinating atoms of the ligand (HL) **a** and their complexes NiL_2_ **b** and ZnL_2_ **c**

Figure 7 shows the optimized molecular structures for the ligand (HL) and its complexes. All geometries correspond to the lowest energy conformations, as determined by density functional theory (DFT) studies. The natural bond orbital (NBO) study of the free ligand reveals that the most negatively charged atoms are O1 (-0.668), N1 (-0.588), N2 (-0.560), N3 (-0.578), and N4 (-0.763), indicating that these could be active donor sites. O1 and N5 are especially well-suited to coordinate with metal ions, allowing the development of stable six-membered chelate rings. When coordinated with Ni(II) and Zn(II), the ligand forms distorted octahedral complexes with six-coordinated metal centers. Atoms N5, O1, N10, and O2 in NiL₂ and ZnL_2_ are essentially in the same plane, with small variations of + 4.327° and + 10.12°, respectively. The N5-O1 distance decreases significantly after complexation, from 3.981 Å in the free ligand to 2.723 Å in the nickel complex and 2.818 Å in the zinc complex (Table 3), indicates the development of strong coordination bonds. The NBO study of the complexes demonstrates electron density redistribution during complex formation. The partial atomic charges of NiL₂ are Ni (+ 0.757), N5 (-0.494), N10 (-0.498), O1 (-0.617), O2 (-0.623), O3 (-0.852), and O4 (-0.855). Charges in ZnL_2_ are as follows: Zn (+ 1.377), N5 (-0.609), N10 (-0.620), O1 (-0.771), O2 (-0.721), O3 (-1.020), and O4 (-0.915). These results demonstrate that the coordination process involves significant charge transfer from donor atoms to metal ions, particularly through nitrogen and oxygen atoms, which improves the stability and electronic delocalization of complexes. Overall, the DFT-optimized structures and NBO charge distribution support the ligand’s great binding affinity for Ni(II) and Zn(II), emphasizing the relevance of specific donor atoms in complex stabilization.

**Table 3:** Selected optimized bond lengths (Å) and bond angles (°) for the complexes NiL_2_ and ZnL_2_

Bond lengths	NiL_2_	ZnL_2_
M1-N5	1.941	2.161
M1-N10	1.948	2.244
M1-O1	1.883	2.066
M1-O2	1.884	2.067
M1-O3	2.508	2.077
M1-O4	2.512	2.299

Angles	NiL_2_	ZnL_2_
N5-M1-O1	90.79	85.42
N5-M1-O2	89.30	93.35
N10-M1-O1	89.41	103.5
N10-M1-O2	90.54	81.53
N1-M1-O2	-	-
O2-M1-O3	-	-
N2-M1-O3	-	-
O3-M1-N5	91.79	84.83
O3-M1-N10	87.82	86.98
O3-M1-O1	92.88	86.99
O3-M1-O2	92.98	102.9
O4-M1-N5	92.68	102.7
O4-M1-N10	87.71	86.38
O4-M1-O1	85.55	72.08
O4-M1-O2	88.58	82.50
N5-M1-N10	179.6	168.9
O1-M1-O2	174.1	153.5
O3-M1-O4	175.3	169.2
N5-O1-N10-O2	+ 4.327*	+ 10.12*
N5-O1-O2-O3	-	-

#### HOMO -LUMO and reactivity parameters

**Fig. 8:**
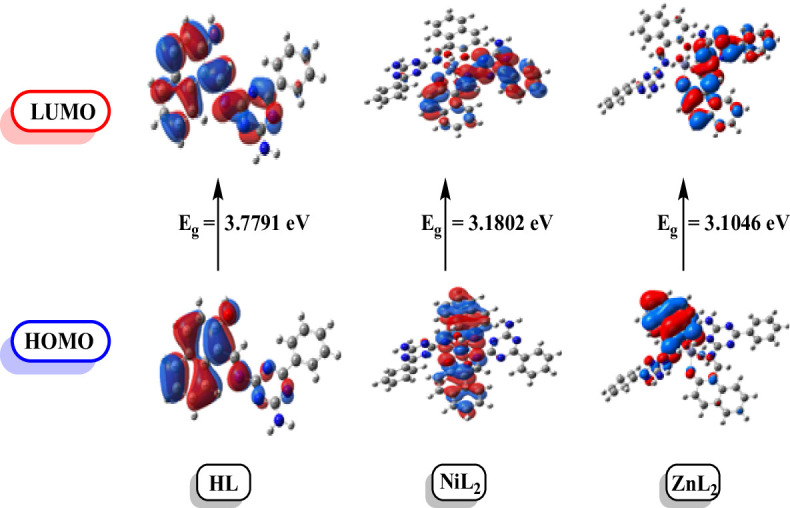
Distributions of HOMO and LUMO electron densities for the ligand (HL) and its metal complexes NiL₂ and ZnL₂

The electronic characteristics of the produced compounds HL, NiL₂, and ZnL₂ were examined using Density Functional Theory (DFT) [6]. Table 4 shows the computed values for total energy, HOMO and LUMO levels, and dipole moments. Metal complexes had larger negative total energy values than free ligands, indicating that they are more thermodynamically stable after coordination with metal ions. Furthermore, compared to HL, the energy gap (Eg = E_LUMO_ - E_HOMO_) decreased in the complexes. Figure 8 shows that the HOMO-LUMO gap in NiL_2_ and ZnL₂ complexes has decreased, indicating better electron delocalization and charge transfer. Higher chemical reactivity and lower kinetic stability are usually linked with smaller gaps, lending support to the idea of increased metal-ligand contact. To further understand electronic reactivity, various global reactivity descriptors were determined, including ionization potential (I), electronegativity (χ), softness (S), chemical potential (µ), global hardness (η), electron affinity (A), and electrophilicity index (ω). The chemical behavior of the compounds is explained by these values, which are based on HOMO and LUMO energies. As can be seen in Table 4, the complexes are more electrophilic and softer than the free ligand, indicating greater reactivity and an improved capacity to function as catalysts or interact with biological systems.

**Table 4:** Calculated energies and properties of ligand HL and complexes NiL_2_, and ZnL_2_

Property	HL	NiL_2_	ZnL_2_
E (a.u.)	-1120.470	-2561.290	-2457.647
HOMO (eV)	-6.1310	-4.9862	-5.3803
LUMO (eV)	-2.3519	-1.8060	-2.2757
E_g_ (eV)	3.7791	3.1802	3.1046
Dipole moment (Debye)	2.7731	2.5988	3.0035
I = -E_HOMO_	6.1310	4.9862	5.3803
A = -E_LUMO_	2.3519	1.8060	2.2757
Χ = (I + A)/2	4.2414	3.3961	3.828
Η = (I -A)/2	1.8895	1.5901	1.5523
S = 1/2η	0.2646	0.3144	0.3221
Μ = -χ	-4.2414	-3.3961	-3.8280
Ω = µ^2^/2η	4.7603	3.6267	4.7199

#### Surface electrostatic potential (MEP) analysis

**Fig. 9:**
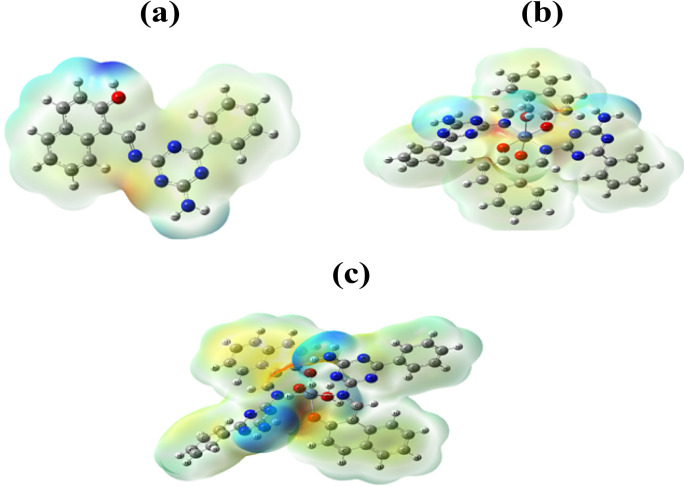
MEP map HL **a**, NiL₂ **b**, and ZnL_2_ **c**

The molecular electrostatic potential (MEP) maps of HL, NiL₂, and ZnL₂ were calculated at the B3LYP/6-311 + + G(d, p) level (Fig. 9). The negatively charged zones (shown in red) are primarily concentrated on electronegative atoms, whereas the positive portions (blue) are frequently connected with hydrogen atoms [6]. This distribution provides information on the likely areas of nucleophilic and electrophilic attack in molecular structures. The results revealed that the oxygen and nitrogen atoms in all configurations have a large negative electrostatic potential, which is related to their strong electronegativity. These negatively charged regions are highlighted in red on the MEP maps and are the most likely targets for nucleophilic attack. In contrast, hydrogen atoms are found in electron-deficient (blue) regions, indicating a lower electron density and potential sites for electrophilic interactions. This surface potential distribution illustrates the electron-drawing behavior of oxygen and nitrogen atoms, which remove electron density from nearby atoms, particularly hydrogen. As a result, regions near electronegative atoms attract electrophilic species, whereas more electron-rich zones are vulnerable to nucleophilic attack. The presence of electron-rich and electron-poor regions explains their ability to interact with biomolecules through hydrogen bonding and electrostatic interactions, which is consistent with the observed DNA-binding and biological activity.

### DNA binding

#### Absorption titration

**Fig. 10:**
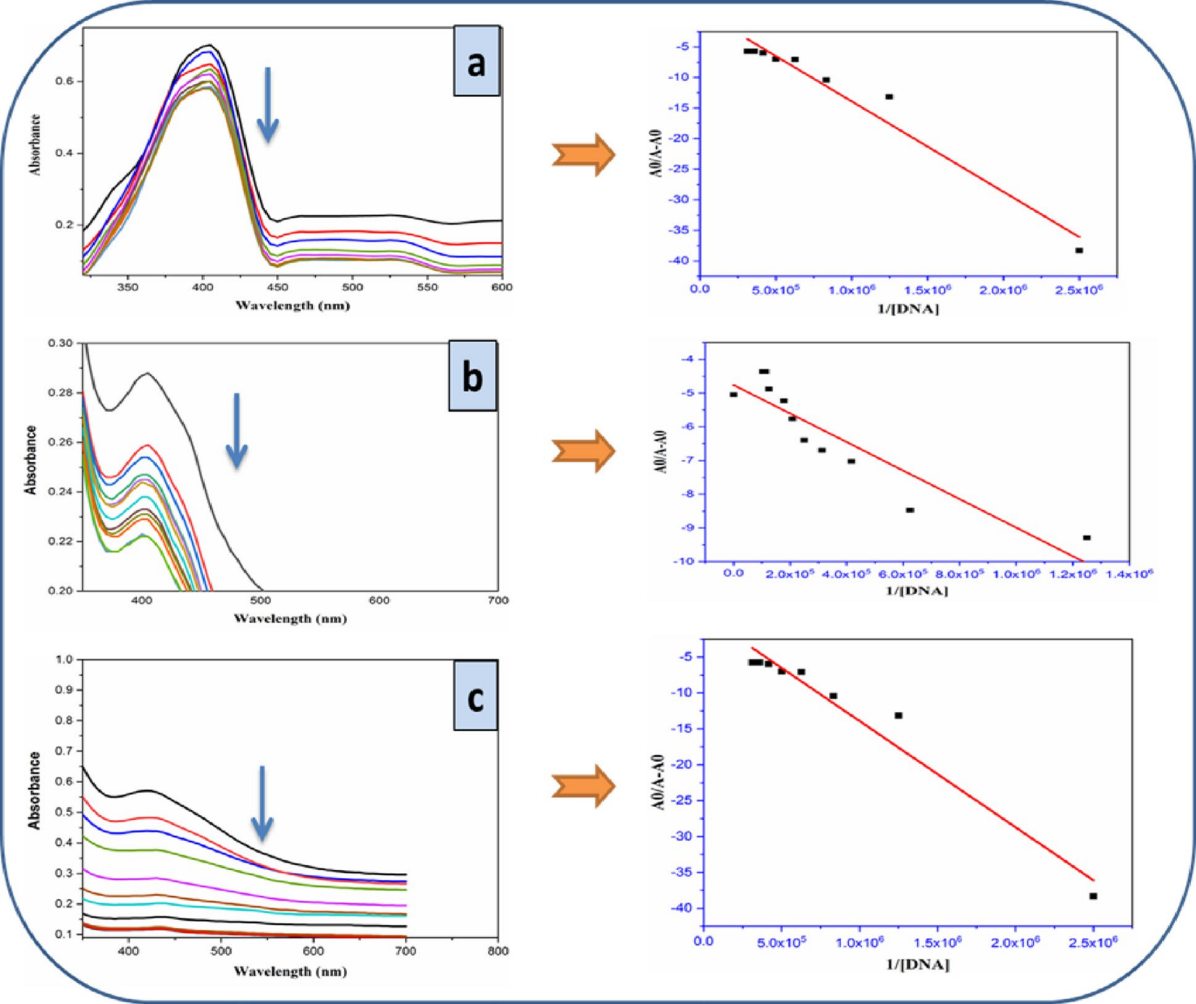
UV–vis spectra and A₀/(A₀ – A) vs. 1/DNA plots for calculating K_b_ of **a** HL, **b** NiL₂, and **c** ZnL₂ with varying CT-DNA concentrations

The ligand, metal complexes, and CT DNA interaction was examined using absorbance titration. The UV-Vis absorption bands varied significantly with DNA concentration: either an increase or decrease in absorbance, and occasionally a minor shift in wavelength (red or blue). These changes provide insight into how metal complexes interact with DNA. Covalent interactions occur when a poorly bound ligand is substituted with a nitrogen base from DNA, namely guanine at the N7 position. Non-covalent interactions take place when base pairs intercalate, engage electrostatically with the phosphate backbone, or bond in the major or minor grooves of the DNA helix [53]. Throughout the experiment, various amounts of CT DNA were added while the studied compounds’ concentration remained constant. UV-vis absorption spectra were used to analyze the interaction of the prepared HL, NiL₂ and ZnL₂ with CT DNA (Fig. 10). Each addition of DNA resulted in a significant hypochromic impact (a drop in absorbance), despite the absence of a noteworthy bathochromic shift (a change in wavelength). According to this characteristic, the binding process may involve partial intercalation rather than traditional groove binding. Based on the spectrum shifts, the Benesi-Hildebrand equation may be used to calculate the binding constant (K_b_) of the compounds with CT DNA (Eq. 1), which helps to assess the strength of interaction [54].

1 $$ \frac{{{\mathrm{A}}_{0} }}{{{\mathrm{A}} - {\mathrm{A}}_{0} }} = \frac{{\varepsilon _{G} }}{{\varepsilon _{{H - G}} - \varepsilon _{G} }} + \frac{{\varepsilon _{G} }}{{\varepsilon _{{H - G}} - \varepsilon _{G} }} \times \frac{1}{{K_{b} \left[ {DNA} \right]}} $$

The absorption coefficients are denoted by ε _G_ and ε _H−G_, while A_0_ and A represent the absorbance values of the free compound and chemical-DNA complex, respectively. (DNA) symbolizes DNA concentration, while K_b_ represents the binding constant. In the graphs of A_0_/A−A_0_ vs. 1/DNA, K_b_ values were calculated by dividing the slope by the y-intercept [55]. The values of the binding constant of the investigated compounds with DNA are 6.0 × 10^4^, 10.1 × 10^5^, and 1.6 × 10^5^ L mol⁻¹ for HL, NiL_2_, and ZnL_2_, respectively. The binding constant value demonstrated that the NiL_2_ complex intercalated with DNA more strongly than the ZnL_2_ complex. Furthermore, Gibbs free energy values (ΔG) were determined using the formula ΔG = − RT ln K_b_, where R is the gas constant and T is the Kelvin temperature. The computations were performed at room temperature. The synthesized compounds had negative ΔG values, indicating spontaneous DNA binding. The NiL_2_ complex demonstrated a better thermodynamic interaction with DNA (-34.22 kJ/mol) compared to HL and ZnL_2_ (-27.24 and − 29.69 kJ/mol) (Table 5).

**Table 5:** DNA binding parameters of HL NiL_2_ and ZnL_2_ estimated from UV–Vis spectral data

Complex	K_b_ (M^− 1^)	ΔG (kJ/mol)
HL	6.0 × 10^4^	-27.24
NiL_2_	10.1 × 10^5^	-34.22
ZnL_2_	1.6 × 10^5^	-29.69

### CV investigation of metal complexes–DNA interactions

**Fig. 11:**
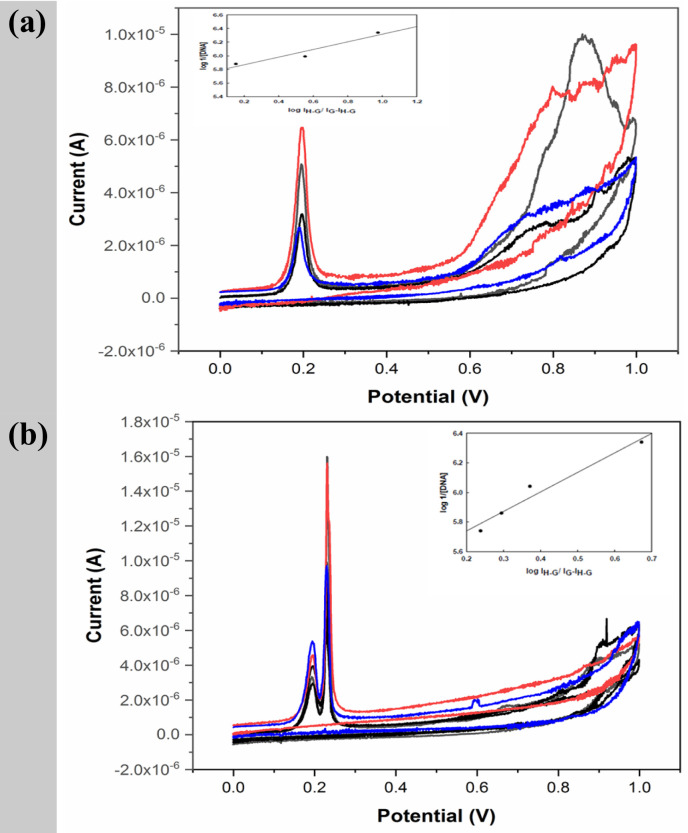
Cyclic voltammograms of 5.96 µM NiL_2_ **a** and ZnL_2_ **b** complexes in the absence and presence of DNA at pH = 7.00. T = 25 °C, t_s_ = 15 s, E_a_ = 0.0 V and scan rate = 100 mV s^− 1^, along with the relationship between the decrease in CV current and DNA concentration

Drug and metal complexes cyclic voltammetry (CV) is used to study DNA binding. Redox processes on the electrode surface may resemble biological ones because electrochemical responses are often equivalent. When a chemical interacts with DNA in CV, its peak potential and current intensity shift. The peak current (Ip) change seen after DNA addition can be used to compute the complex-DNA binding constant. Any peak potential shift implies a compound-DNA binding mechanism. CV methods provide a useful complement in support of UV–visible. The electrochemical behavior of the synthesized NiL_2_ and ZnL_2_ complexes was investigated using cyclic voltammetry in the absence and presence of CT-DNA (Fig. 11(a & b)). In the free NiL_2_ complex solution, a well-defined anodic peak was observed at approximately 0.20 V, indicating the oxidation of the electroactive center of the complex. Upon incremental addition of CT-DNA, a progressive decrease in the anodic peak current (I_p.a._) was recorded, while the anodic peak potential (E_p.a._) remained essentially unchanged.

In contrast, the voltammogram of the ZnL_2_ complex in the presence of DNA revealed two distinct anodic peaks at approximately 0.20 V and 0.23 V, as opposed to a single anodic peak in the NiL_2_ complex. This splitting suggests either slow oxidation processes or the presence of several binding mechanisms between the zinc complex and DNA (such as intercalative and groove binding). The twofold peak pattern may potentially reflect various electronic environments inside the complex following DNA binding, which could be related to conformational changes or the ligand’s partial involvement in redox activity.

This decrease in current is attributed to the formation of a complex–DNA adduct, which reduces the diffusion of free electro-active species toward the electrode surface, thereby suppressing electron transfer. However, the absence of a significant shift in the peak potential suggests that the electronic environment around the metal center is not substantially altered upon DNA binding. The observed decrease in current intensity without a significant shift in potential suggests that the complexes interact with DNA primarily through partial intercalation.

The binding constant of the complex-DNA adduct can be calculated using the following formula, which is based on changes in peak height (I_p_) of the generated complex after DNA addition (Eq. 2) [56]:

2 $$ \frac{{{\mathrm{A}}_{0} }}{{{\mathrm{A}} - {\mathrm{A}}_{0} }} = \frac{{\varepsilon _{G} }}{{\varepsilon _{{H - G}} - \varepsilon _{G} }} + \frac{{\varepsilon _{G} }}{{\varepsilon _{{H - G}} - \varepsilon _{G} }} \times \frac{1}{{K_{b} \left[ {DNA} \right]}} $$

Here, I_G_ and I_H–G_ denote the peak currents of the free guest (G) and the complex (H–G), respectively, while K refers to the apparent binding constant. When plotting log(1/DNA) against log[I_H–G_/(I_G_ – I_H–G_)], a linear relationship is obtained, with the intercept corresponding to log K. The calculated binding constants for the NiL_2_ and ZnL_2_ complexes were 5.69 × 10^5^ M^− 1^ and 2.51 × 10^5^ M^− 1^, respectively (Table 6). These statistics indicate that there is a relatively good interaction with DNA. The remarkable agreement between binding constants determined by UV-Vis spectroscopy and those obtained by cyclic voltammetry indicated the consistency and dependability of the interaction data.

**Fig. 12:**
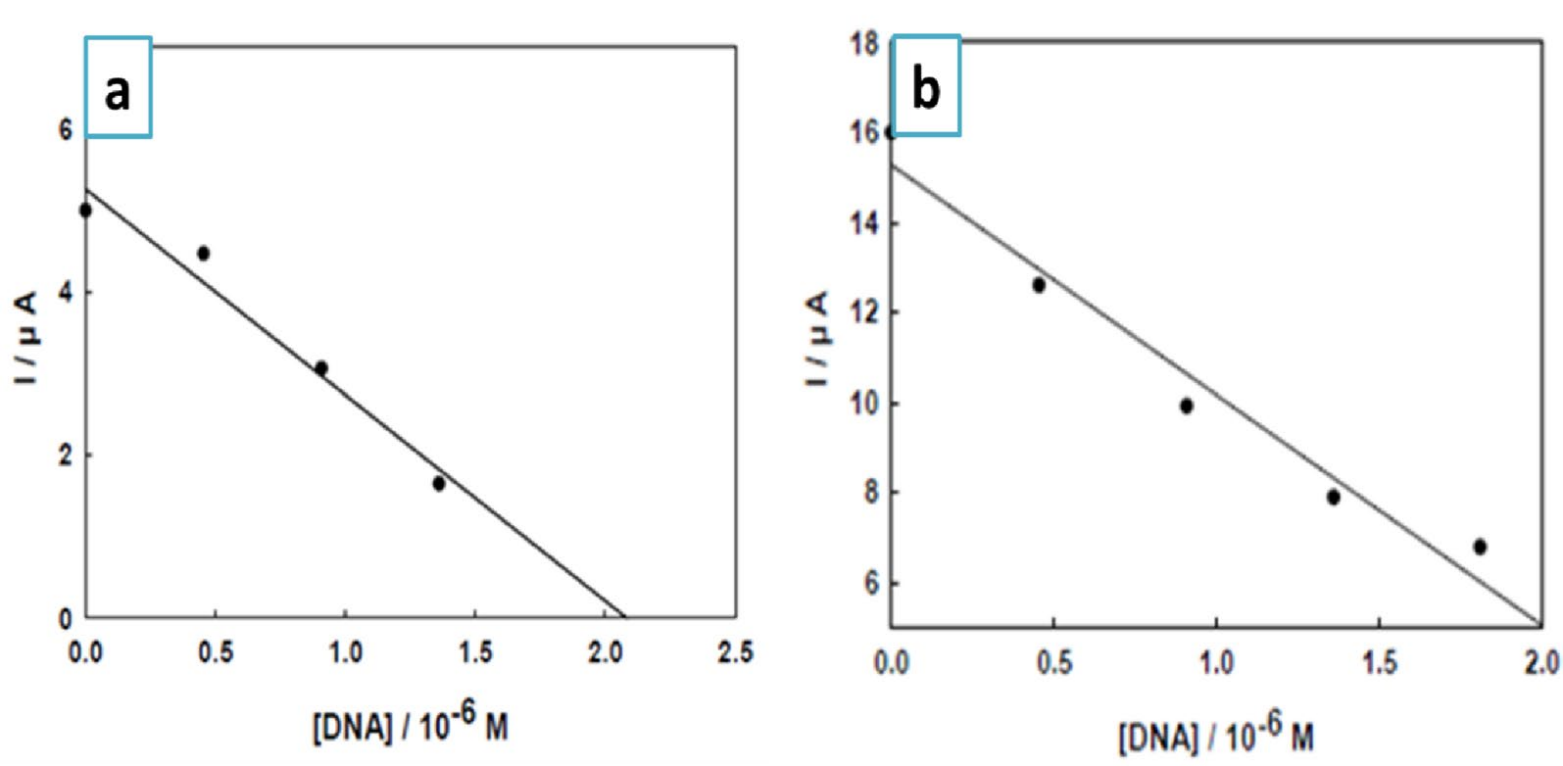
Relationship between the decrease of CV current of 5.96 µM NiL_2_ **a**, ZnL_2_ **b** complexes and the concentration of DNA at pH 7.0

The addition of DNA progressively reduces the peak current of the NiL₂ and ZnL₂ complexes, as illustrated in Fig. 12(a & b). This enables the calculation of DNA concentration at pH 7.0. To assess the method’s sensitivity, the limits of detection (LOD) and limit of quantification (LOQ) were computed using the calibration curve acquired at four distinct time intervals. The following standard Eqs. (3 & 4) were used for the calculations [57]:

3 $$ {\mathbf{LOD}}\, = \,{\mathbf{3}}{\text{ }}{\mathbf{S}}.{\mathbf{D}}/{\mathbf{b}} $$

4 $$ {\text{LOQ = 10}}\,\,{\mathrm{S}}{\text{.D / b }} $$

The slope of the calibration curve is represented by b, while the standard deviation of the intercept is denoted by S.D. Table 7 displays the calculated values. These results suggest that the Ni complex has the potential to be used as a novel electrochemical indicator for the monitoring of DNA concentration. The proposed technique is optimal for the evaluation of DNA levels in clinical samples due to its simplicity, sensitivity, and rapidity.

**Table 6:** Binding constants NiL_2_ and ZnL_2_ nano complexes with DNA (pH 7.0) at 298 K

System	Straight line equation I(µA) = a + bC	Regression coefficient (*R*)	K (10^5^ M^− 1^)
Ni complex -DNA	Y = 5.7554 + 0.5621X	0.920	5.69
Zn complex -DNA	Y = 5.4749 + 1.3213X	0.980	2.51

**Table 7:** Calibration curve data for the determination of DNA in presence of NiL_2_and ZnL_2_ complexes

System	Straight line equation I(µA) = a + bC	Regression coefficient (*R*)	LOD (M)	LOQ (M)
Ni complex -DNA	Y = 5.2644-2.5272 × 10^6^ X	0.965	1.94 × 10^− 7^	6.49 × 10^− 7^
Zn complex -DNA	Y = 15.2758-5.1167 × 10^6^ X	0.982	1.57 × 10^− 7^	5.23 × 10^− 7^

#### Investigation of DNA interaction mechanism

**Fig. 13:**
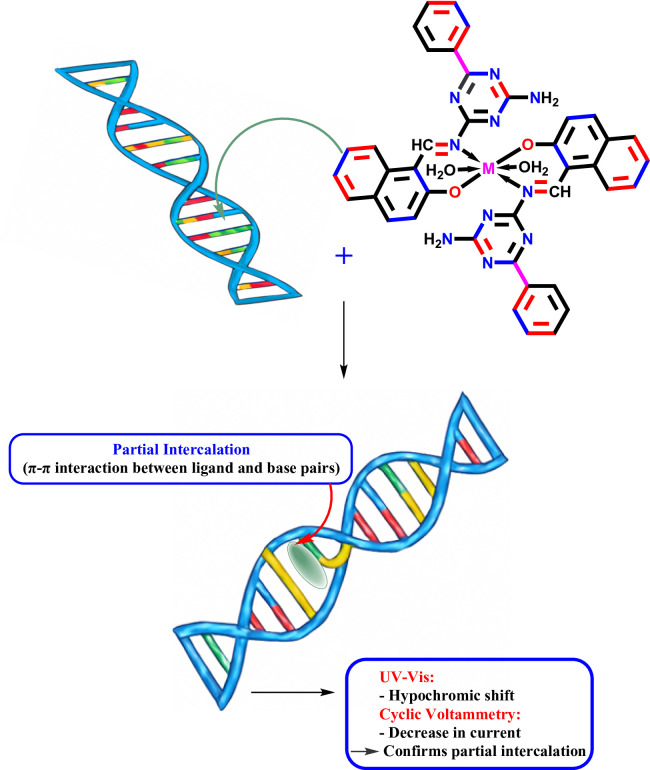
DNA binding mechanism of HL ligand and its NiL₂ and ZnL₂ complexes through partial intercalation

Metal complexes can bind to DNA by intercalation, replacement, or partial intercalation mechanisms depending on the nature of the ligand and metal center [58]. Understanding these types of interactions is required when investigating the biological activity of metal-based drugs utilized in antibiotics and chemotherapy. We used cyclic voltammetry (CV) and UV-Vis spectroscopy to explore the prepared compounds’ interactions with calf thymus DNA (CT-DNA), as seen in Fig. 13. The UV spectra showed hypochromic shifts, and the CV results showed changes in the current intensity, which together suggest a partial intercalation binding. In this case, the flat part of the ligand slides between the DNA base pairs, while other parts of the molecule may interact through electrostatic attraction or groove binding. This type of interaction allows π–π stacking between the complex and DNA bases, leading to moderate binding strength. Metal ions (Ni^2+^ and Zn^2+^) in the complexes may increase their viscosity by coordinating with the DNA phosphate groups or by increasing their stability through hydrogen bonds and van der Waals forces. All these observations indicate that the interaction is not only an intercalation interaction, but also involves a mixed or partial intercalation process.

#### Antimicrobial activity

**Fig. 14:**
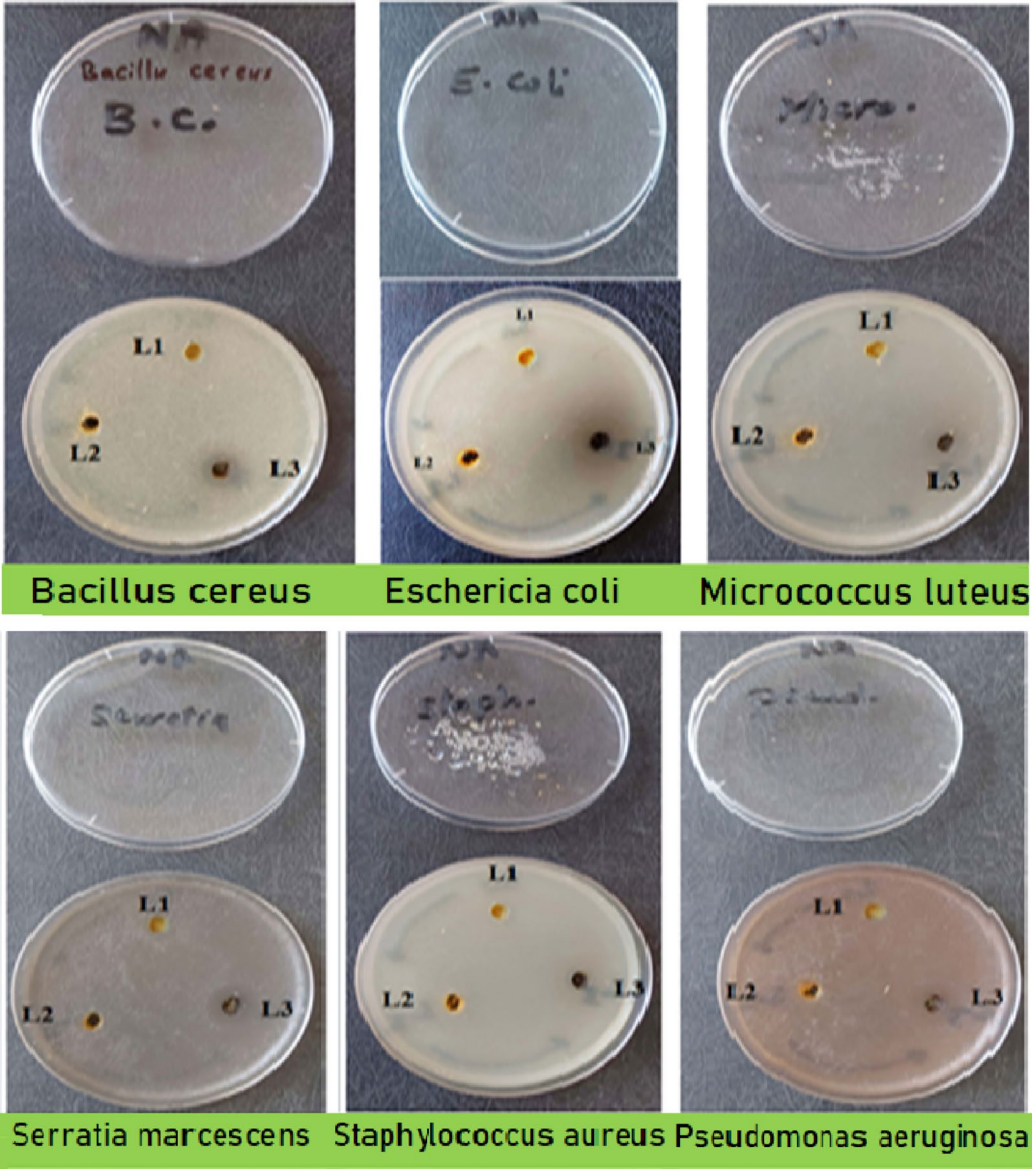
Inhibition zones produced by the synthesized HL, NiL₂, and ZnL₂ against selected bacterial strains

The newly produced Schiff base, designated as HL, along with its corresponding metal chelates, was subjected to rigorous evaluation for its antimicrobial efficacy against a total of six pathogenic bacterial strains, specifically comprising three Gram-negative organisms (*E. coli*,* P. aeruginosa*,* and S. marcescens*) and three Gram-positive organisms (*B. cereus*,* M. luteus*,* and S. aureus*), in addition to six pathogenic fungal species, namely *A. flavus (Af)*,* S. brevicaulis (Sb)*,* F. oxysporum (Fo)*,* C. albicans (Ca)*,* G. candidum (Gc)*,* and T. rubrum (Tr)*, employing the disk diffusion methodology (Table 8; Fig. 14). The obtained data were juxtaposed with standard antibiotics, specifically chloramphenicol (CHL), which served as the antibacterial benchmark. Furthermore, clotrimazole (CLO) served as a standard reference for antifungal activity. Experimental results in vitro indicated that the complexes were more effective against bacteria and fungi than the uncoordinated ligand. As explained by Tweedy’s theory [50], the chelation process diminishes the polarity of metal ions by partial sharing of their positive charge with donor atoms and through possible delocalization of π-electrons across the chelate framework [30]. This improvement in activity supports this theory. Accordingly, the higher activity of the metal complexes relative to the free ligand can be explained by the chelation effect within the studied series. As a result, the complex becomes more lipophilic, allowing it to penetrate through the lipid layers of microbial cell membranes and block metal-binding sites on essential enzymes, eventually impairing the microorganism’s function [59, 60]. Additional factors that may enhance the antimicrobial activity include solubility, conductivity, and the bond length between the metal and the ligand. Among all the complexes evaluated, the Zn(II) complex exhibited the most pronounced antibacterial activity against the tested bacteria and fungi; conversely, HL demonstrated no significant activity against the assessed bacterial strains.

**Table 8:** Antimicrobial activity of the tested compounds expressed as inhibition zone diameter (mm)

Bacteria	Compound			
	HL	NiL_2_	ZnL_2_	CHL
*E. coli (G-ve)*	0	10	12	28
*M. luteus (G + ve)*	0	7	12	15
*P. aeruginosa (G-ve)*	0	10	13	18
*S. aureus (G + ve)*	0	7	11	16
*S. marcescens (G-ve)*	0	7	12	15
*B. cereus (G + ve)*	0	0	12	26
*T. rubrum*	12	16	34	42
*C. albicans*	7	7	16	18
*G. candidum*	0	0	17	18
*S. brevicaulis*	0	0	16	22
*F. oxysporum*	0	14	14	18

Fungi	Compound			
	HL	NiL_2_	ZnL_2_	CLO
*A. flavus*	0	0	10	28
*T. rubrum*	12	16	34	42
*C. albicans*	7	7	16	18
*G. candidum*	0	0	17	18
*S. brevicaulis*	0	0	16	22
*F. oxysporum*	0	14	14	18

#### In vitro cytotoxic evaluation

**Fig. 15:**
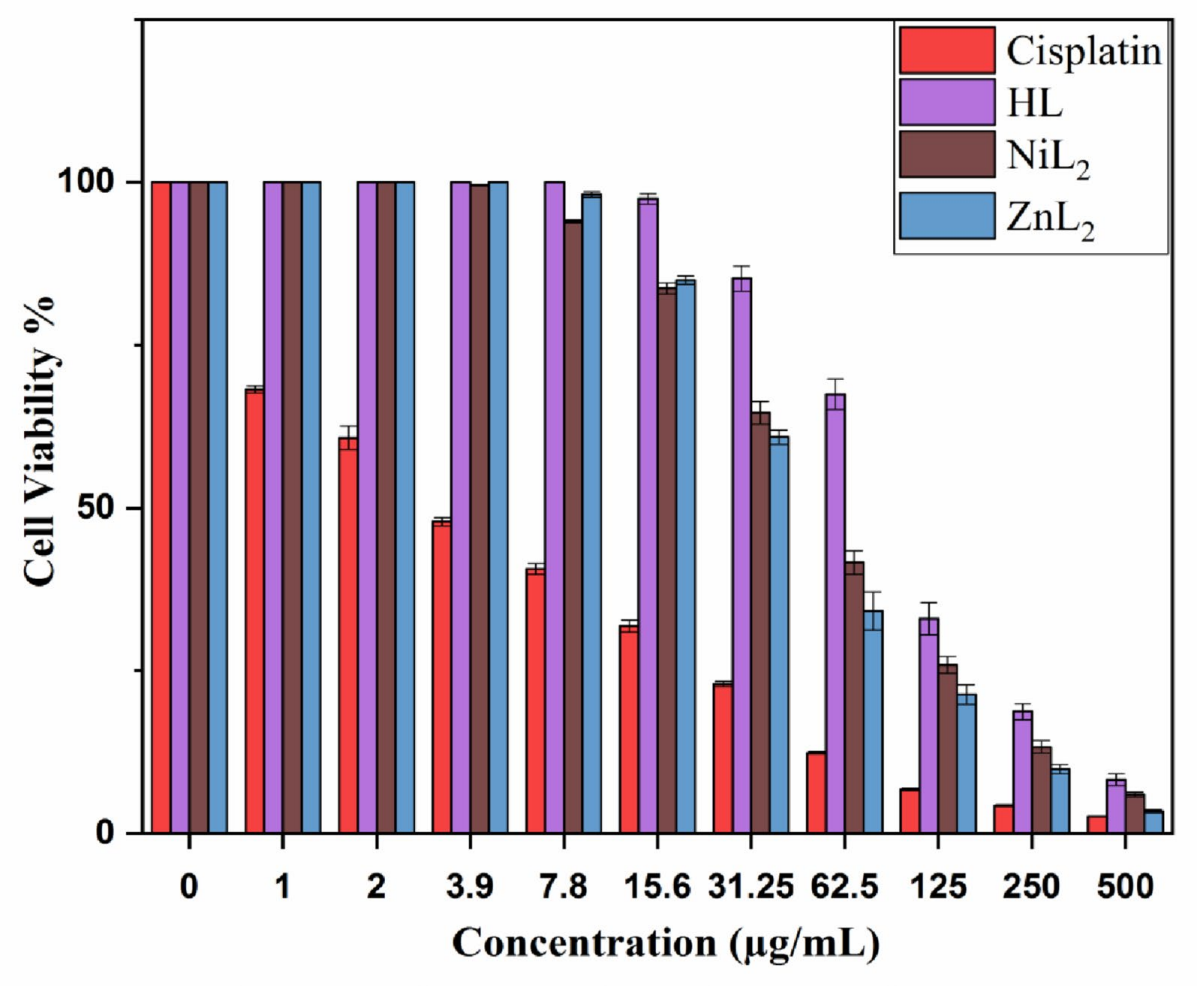
Viability of HepG-2 cells treated with cisplatin, HL, NiL_2_, and ZnL_2_ at different concentrations

Metal complexes are attractive scaffolding for creating new anticancer drugs due to their ability to obtain low IC₅₀ values while minimizing negative effects [61]. The cytotoxic potential of the synthesized compounds was evaluated using the MTT assay, and the results are presented in Table 9 and shown in (Fig. 15). The compounds were evaluated at doses ranging from 1 to 500 µg/mL and cell viability and growth inhibition percentages were measured (Tables 10, 11, 12 and 13). Cytotoxicity was measured by calculating the percentage of cell growth inhibition compared to an untreated control group with 100% growth. The results showed that the chemical type and concentration of the investigated substances had a substantial impact on the level of inhibition, demonstrating a different concentration-dependent response. Furthermore, the cytotoxic activity of the complexes increased with increasing concentrations, supporting the dose-dependent pattern. ZnL₂ exceeded NiL₂ with regard to cytotoxicity against the HepG-2 liver cancer cell line. The free ligand (H_2_L), NiL_2_, and ZnL_2_ complexes exhibited IC_50_ values of 94.10, 51.08, and 43.94 µg/mL, respectively, while the reference medication, cisplatin, showed a significantly lower IC_50_ of 3.58 µg/mL. Previous study indicates that metal-based compounds have superior cytotoxic capabilities against cancer cells [62]. One possible mechanism is the induction of cell cycle arrest during the S phase, which is commonly associated with enhanced expression of tumor-suppressor genes [63]. In addition, the anticancer effect might arise from the complexes’ capacity to bind with DNA as well as proteins, indicating their potential as multi-target therapeutic agents. Furthermore, density functional theory (DFT) calculations revealed that ZnL_2_ possesses a high dipole moment, suggesting enhanced biological activity. These computational findings align well with the experimental results.

**Table 9:** Cytotoxic potential of HL ligand and associated metal complexes on HepG-2 liver cancer cells

Cell line	Median inhibitory concentration (IC_50_) in µg/mL			
	HL	NiL_2_	ZnL_2_	Cisplatin
HepG-2	94.10 ± 2.87	51.08 ± 1.23	43.94 ± 2.16	3.58 ± 0.26

**Table 10:** Cell viability (%) of HepG-2 cells treated with Cisplatin at various concentrations, as measured by MTT assay

Sample conc. (µg/ml)	Viability %	S.D. (±)
500	2.58	0.06
250	4.31	0.13
125	6.75	0.21
62.5	12.39	0.18
31.25	22.98	0.41
15.6	31.87	0.91
7.8	40.62	0.86
3.9	47.89	0.67
2	60.75	1.83
1	68.17	0.54
0	100	

**Table 11:** Viability and inhibition percentages of HepG-2 cells treated with HL at different concentrations, as determined by MTT assay

Sample conc. (µg/ml)	Viability %	Inhibitory %	S.D. (±)
500	8.27	91.73	0.92
250	18.70	81.3	1.24
125	32.98	67.02	2.46
62.5	67.41	32.59	2.33
31.25	85.23	14.77	1.95
15.6	97.46	2.54	0.82
7.8	100	0	
3.9	100	0	
2	100	0	
1	100	0	
0	100	0	

**Table 12:** Viability and inhibition percentages of HepG-2 cells treated with NiL_2_ at different concentrations, as determined by MTT assay

Sample conc. (µg/ml)	Viability %	Inhibitory %	S.D. (±)
500	5.96	94.04	0.42
250	13.29	86.71	0.95
125	25.87	74.13	1.31
62.5	41.60	58.4	1.82
31.25	64.59	35.41	1.73
15.6	83.69	16.31	0.84
7.8	94.03	5.97	0.21
3.9	99.56	0.44	0.12
2	100	0	
1	100	0	
0	100	0	

**Table 13:** Viability and inhibition percentages of HepG-2 cells treated with ZnL_2_ at different concentrations, as determined by MTT assay

Sample conc. (µg/ml)	Viability %	Inhibitory %	S.D. (±)
500	3.41	96.59	0.23
250	9.85	90.15	0.71
125	21.36	78.64	1.48
62.5	34.17	65.83	2.95
31.25	60.83	39.17	1.12
15.6	84.95	15.05	0.63
7.8	98.12	1.88	0.44
3.9	100	0	
2	100	0	
1	100	0	
0	100	0	

#### Molecular docking evaluation

**Fig. 16:**
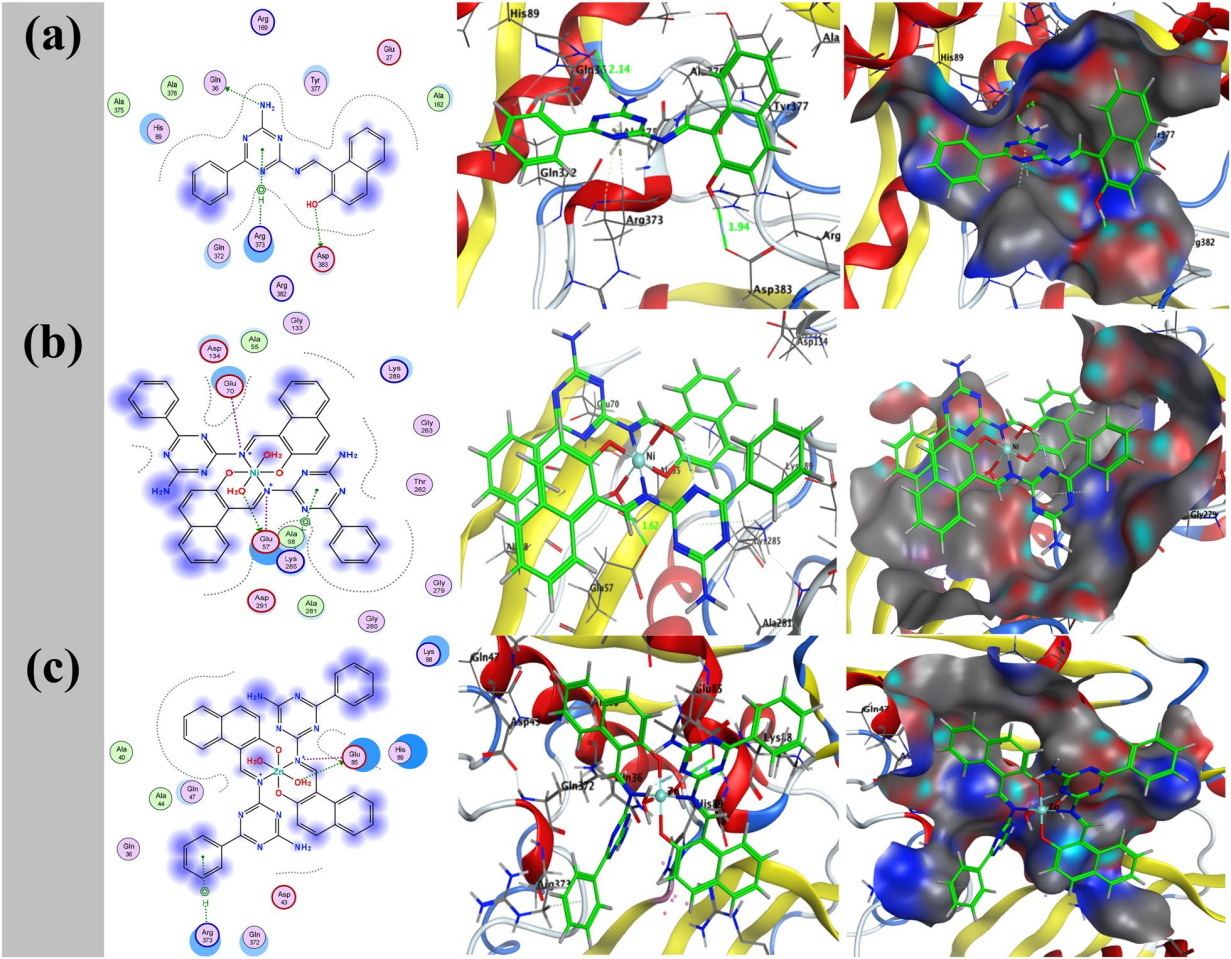
2D and 3D representations of HL with NiL₂ **a** and ZnL₂ **b** bound to the liver cancer receptor (PDB ID: 5A19). Hydrophobic interactions are indicated by dotted lines

**Fig. 17:**
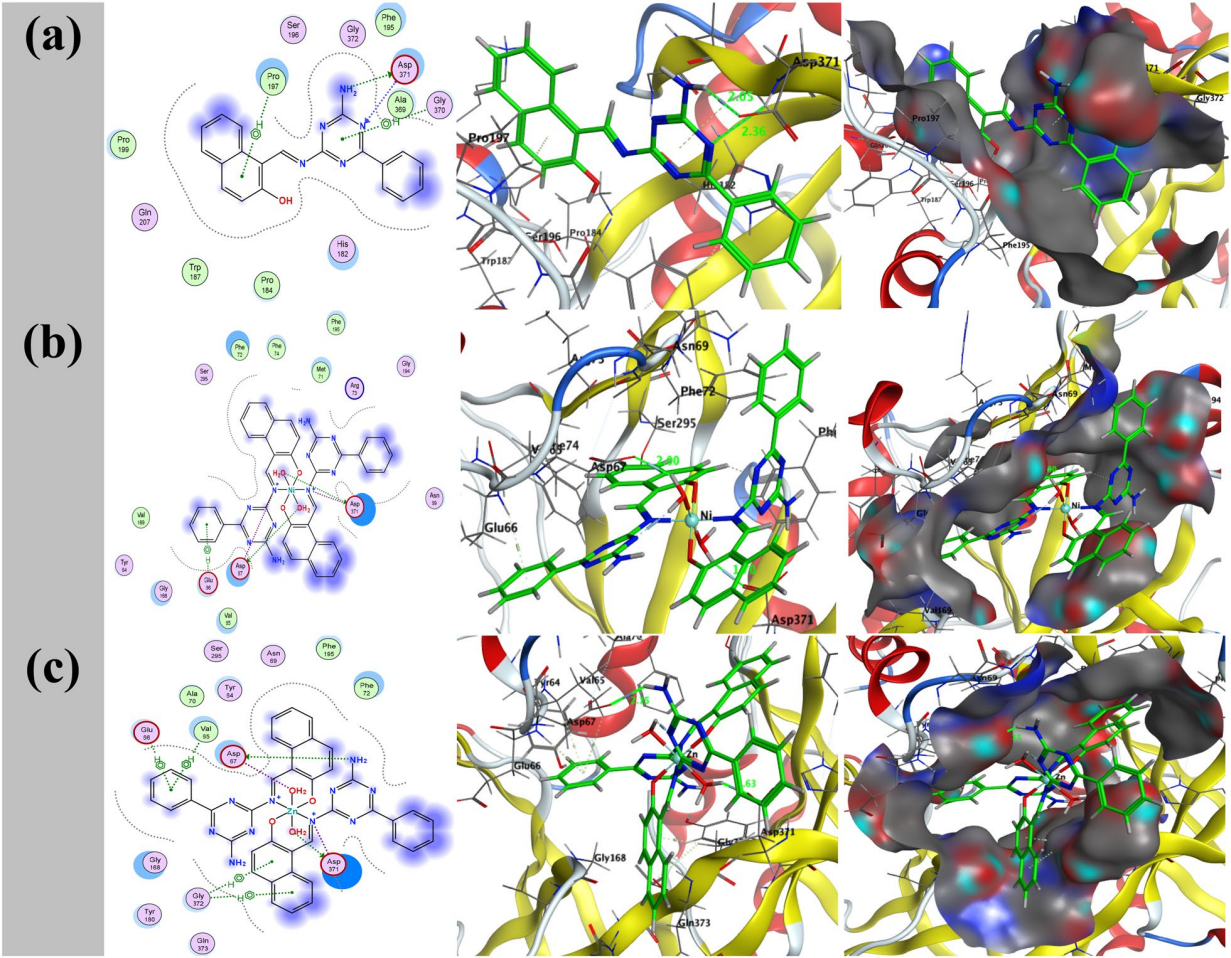
2D and 3D representations of HL with NiL₂ **a** and ZnL₂ **b** bound to the *A. flavus* receptor (PDB ID: 8HBS). Hydrophobic interactions are indicated by dotted lines

Molecular docking is a powerful computational tool for investigating atomic-level interactions between ligands and their protein targets. This approach aids in understanding how small molecules operate within protein active regions while also shedding information on critical metabolic pathways. Metal complexes formed from ligands have recently received interest due to their potent anticancer activities against a wide range of cancer types [64]. In addition to their cytotoxic effects, numerous metal-based compounds have demonstrated promising antifungal capabilities, particularly against dangerous fungus such as *A. flavus*, via interfering with gene regulatory pathways [65]. The present study involved docking studies of the free ligand HL and its metal complexes NiL₂ and ZnL₂ against two distinct protein targets: a gene regulation receptor from liver cancer (PDB ID: 5A19), and *A. flavus* (PDB ID: 8HBS) as illustrated in (Figs. 16 and 17). Table 10 displays the docking data, which include binding scores and the amino acid residues that were implicated. The computed binding free energy for the liver cancer receptor (5A19) were − 8.0 kcal/mol for the HL receptor, -42.8 kcal/mol for the NiL₂ receptor, and − 39.4 kcal/mol for the ZnL₂ receptor. The binding energies of the *A. flavus* receptor (8HBS) were discovered to be -4.5, -50.2, and − 46.3 kcal/mol, respectively. ZnL₂ had the lowest binding energy values, indicating the highest contact with target proteins. Stronger and more persistent ligand-protein interactions are typically associated with larger negative binding energies. Thus, the evidence points to ZnL₂ as a potentially effective therapeutic agent, especially for the treatment of liver cancer. The accuracy of the docking results is further improved by the agreement between computational predictions and experimental measurements.

**Table 14:** Docking results of HL, NiL₂, and ZnL₂ with liver cancer (5A19) and *Aspergillus flavus* (8HBS) targets, including binding affinities and critical interactions

Target	Compound	Receptor	Interaction	Distance(Å)*	E (kcal/mol)
(PDB ID: 5A19)	HL				
	N 13	OE1 GLN 36	H-donor	3.04 (2.14)	-0.6
	O 26	OD2 ASP 383	H-donor	2.87 (1.94)	-5.2
	6-ring	CA ARG 373	pi-H	3.94	-1.7
	6-ring	CG ARG 373	pi-H	4.32	-0.5
	NiL_2_				
	O 85	OE2 GLU 57	H-donor	2.64 (1.62)	-30.1
	N 14	OE1 GLU 70	Ionic	3.79	-1.0
	N 55	OE2 GLU 57	Ionic	3.24	-3.1
	O 85	OE2 GLU 57	Ionic	2.64	-7.4
	6-ring	NZ LYS 285	pi-cation	4.64	-1.2
	ZnL_**2**_				
	O 85	OE2 GLU 85	H-donor	2.72 (1.73)	-27.2
	N 55	OE2 GLU 85	Ionic	3.25	-3.0
	O 85	OE2 GLU 85	Ionic	2.72	-8.6
	6-ring	CD ARG 373	pi-H	3.63	-0.6
(PDB ID: 8HBS)	HL				
	N 13	OD2 ASP 371	H-donor	3.03 (2.05)	-2.1
	N 12	N ASP 371	H-acceptor	3.28 (2.36)	-0.5
	6-ring	CD PRO 197	pi-H	3.72	-0.5
	6-ring	CA GLY 370	pi-H	3.80	-0.5
	NiL_2_				
	O 82	OD2 ASP 67	H-donor	2.76 (2.00)	-7.0
	O 85	OD1 ASP 371	H-donor	2.69 (1.70)	-20.3
	N 14	OD1 ASP 67	Ionic	3.96	-0.6
	N 14	OD2 ASP 67	Ionic	3.73	-1.1
	N 55	OD1 ASP 371	Ionic	3.29	-2.8
	N 55	OD2 ASP 371	Ionic	3.31	-2.7
	O 82	OD2 ASP 67	Ionic	2.76	-6.3
	O 85	OD1 ASP 371	Ionic	2.69	-7.0
	6-ring	N GLU 66	pi-H	3.91	-2.4
	ZnL_2_				
	N 54	OD2 ASP 67	H-donor	3.08 (2.15)	-5.1
	O 85	OD1 ASP 371	H-donor	2.60 (1.63)	-21.8
	N 55	OD1 ASP 371	Ionic	3.68	-1.3
	O 82	OD1 ASP 67	Ionic	3.33	-2.6
	O 82	OD2 ASP 67	Ionic	3.11	-3.8
	O 85	OD1 ASP 371	Ionic	2.60	-7.7
	6-ring	CA VAL 65	pi-H	4.14	-0.9
	6-ring	CG1 VAL 65	pi-H	4.30	-0.6
	6-ring	N GLU 66	pi-H	3.86	-0.6
	6-ring	CA GLY 372	pi-H	4.52	-0.5
	6-ring	CA GLY 372	pi-H	3.72	-0.9

## Conclusion

The present study reports the eco-friendly synthesis of a new Schiff base ligand (HL) and its NiL₂ and ZnL₂ complexes using a green chemistry approach. The synthesized metal complexes showed strong interaction with CT-DNA, suggesting a partial intercalative binding mode. This interaction is consistent with the observed cytotoxic activity of the complexes. Compared to the free ligand, the metal complexes exhibited higher antibacterial and antifungal activities. In addition, the complexes showed cytotoxic effects against HepG-2 cell lines. The experimental findings were supported by molecular docking studies. These results indicate that the prepared metal complexes possess multiple biological activities and may be of interest for further studies in medicinal chemistry.

## Data Availability

All data generated or analyzed during this study are included in this article and the raw data is available from the corresponding author if it requested.
